# Tacrolimus (FK506) Suppresses TREM-1 Expression at an Early but Not at a Late Stage in a Murine Model of Fungal Keratitis

**DOI:** 10.1371/journal.pone.0114386

**Published:** 2014-12-02

**Authors:** Weilan Huang, Shiqi Ling, Xiuhua Jia, Binwu Lin, Xi Huang, Jing Zhong, Weihua Li, Xiaolei Lin, Yifang Sun, Jin Yuan

**Affiliations:** 1 State Key Laboratory of Ophthalmology, Zhongshan Ophthalmic Center of Sun Yat-sen University, Guangzhou, China; 2 Department of Ophthalmology, The Third Affiliated Hospital of Sun Yat-Sen University, Guangzhou, China; 3 Physical Examination Center, The Third Affiliated Hospital of Sun Yat-Sen University-Lingnan Hospital, Guangzhou, China; Singapore Immunology Network, Singapore

## Abstract

**Purpose:**

To investigate the efficacy and mechanism of tacrolimus(FK506), which is a novel macrolide immunosuppressant, in inhibiting triggering receptor expressed on myeloid cells-1 (TREM-1) expression in a murine keratitis model induced by *Aspergillus fumigatus.*

**Method:**

TREM-1 was detected in 11 fungus-infected human corneas by quantitative real-time PCR (qRT-PCR). RAW264.7 macrophages were divided into four groups, which received treatment with zymosan (100 µg/ml), zymosan (100 µg/ml) + mTREM-1/Fc protein (1 µg/ml), or zymosan (100 µg/ml) + FK506 (20 µM) or negative-control treatment. After this treatment, the expression of TREM-1, interleukin-1β (IL-1β) and tumor necrosis factor α (TNFα) was assayed using qRT-PCR and ELISA. The mouse model of fungal keratitis was created by intrastromal injection with *Aspergillus fumigatus*, and the mice were divided into 2 groups: group A received vehicle eye drops 4 times each day, and group B received 4 doses of FK506 eye drops each day. Corneal damage was evaluated by clinical scoring and histologic examination,and myeloperoxidase (MPO) protein levels were also detected by ELISA. The expression of TREM-1, IL-1β and TNFα was then determined at different time points using qRT-PCR and ELISA.

**Results:**

TREM-1 expression dramatically increased in the human corneas with fungal keratitis. In contrast, FK506 reduced the expression of TREM-1, IL-1β and TNFα in RAW264.7 macrophages stimulated with zymosan. In the mouse model, at day 1 post-infection, the corneal score of the FK506-treated group was lower than that of the control, and polymorphonuclear neutrophil (PMN) infiltration was diminished. TREM-1, IL-1β and TNFα expression was significantly reduced at the same time point. However, the statistically significant differences in cytokine expression, clinical scores and infiltration disappeared at 5 days post-infection.

**Conclusions:**

FK506 may inhibit the inflammation induced by fungi and alleviate the severity of corneal damage at an early stage of fungal keratitis by downregulating TREM-1 expression.

## Introduction

Fungal keratitis is a sight-threatening ocular disease with a growing incidence, especially in developing countries [1]. The pathogens underlying fungal keratitis are varied due to differences in climates and economic environments. In China, the most common pathogens are *Fusarium solani* and *Aspergillus fumigatus* [2]. The immune response to these infectious microorganisms includes both adaptive immunity and innate immunity. Neutrophils are active inflammatory immune cells in innate immunity, quickly arriving at a lesion to eliminate fungi at an early stage [3]. Many studies have confirmed that macrophages also play an important role, mediating the acquired immune response to eradicate infection [4], usually at a later stage of infection. However, excessive inflammation due to not only adaptive immunity but also innate immunity can cause tissue damage and even life-threatening consequences. In fact, inflammation is likely one of the most important causes of corneal destruction after fungal infection because infected corneas often undergo a serious suppurative process [5]. In the present study, a test for myeloperoxidase (MPO) protein was used to detect infiltrating neutrophils over the short time course of an *Aspergillus fumigatus*-induced keratitis model. In addition, macrophages were used in an in vitro study.

Triggering receptor expressed on myeloid cells-1 (TREM-1) is a newly identified receptor that belongs to the Ig superfamily. This receptor is highly expressed on the surface of granulocytes and a subset of monocyte/macrophages [6]. Although the natural ligand of TREM-1 remains unknown, experiments using TREM-1-agonist monoclonal antibodies indicate that TREM-1 engagement can stimulate the production of certain proinflammatory cytokines, such as tumor necrosis factor α (TNFα) and interleukin (IL)-1β [7]–[9]. It is also known that TREM-1 expression levels are highly increased in different tissues infected by bacteria [10] or fungi [11]. Thus, the blockade of TREM-1 with a soluble mTREM-1/IgG fusion protein reduces the TREM-1-mediated inflammatory response and the severity of infectious diseases, such as *Pseudomonas aeruginosa*-related keratitis [10], septic shock [6] and inflammatory bowel disease (IBD) [9], [12]. The studies cited above established that TREM-1 is involved in inflammation and is a suitable candidate to target to reduce inflammation and alleviate the severity of inflammatory diseases, including those in the cornea. Further studies suggested that TREM-1 acts synergistically with Toll-like receptors (TLRs) and Nod-like receptors to amplify proinflammatory responses [6], [13], which indicates that TREM-1 amplifies inflammation.

Macrolides are primarily antibiotics and are generally used to treat infections caused by Gram-positive bacteria, rickettsiae, chlamydiae, *Mycoplasma pneumoniae* and certain Gram-negative bacteria [14], [15]. Recent studies have demonstrated that macrolide antibiotics, such as roxithromycin, clarithromycin, erythromycin, and azithromycin, also possess anti-inflammatory properties in addition to their antimicrobial ability [16]. Tacrolimus (FK506), a macrolide molecule, was initially isolated as an antifungal compound, and a previous report demonstrated that FK506 is relatively active against *Aspergillus fumigatus* [17]. Further investigation demonstrated that TREM-1 is also a potent immunosuppressant; it is thus widely used to avoid the rejection of solid-organ allografts and to treat autoimmune diseases [18]. Moreover, the potency of FK506 is 50- to 100-fold higher than that of cyclosporine A (CsA) [19]. Clinicians tend to use FK506 as an immunosuppressant due to its limited antifungal ability. It has been demonstrated that the anti-inflammatory capacity of FK506 can affect various components of the inflammatory cascade, such as inhibiting neutrophil infiltration [20], reducing the expression of TNFα by inhibiting the activation of microglia in vitro [21], [22] and suppressing the release of IL-1α and TNFα from macrophages [23], [24]. In a mouse model of pleurisy, FK506 also exhibited potent anti-inflammatory properties by inhibiting the expression of proinflammatory cytokines (TNFα and IL-1β), reducing the activity of enzymes (MPO and adenosine deaminase) and down-regulating the levels of inflammatory mediators (bradykinin, histamine and substance P) [25].

In the present study, we tested the effect of FK506 on alleviating inflammation in the cornea and examined TREM-1 expression as a proxy for inflammation severity in mouse macrophages and corneas.

## Materials and Methods

### Patients and sample collection

Clinical samples were surgically removed at Zhongshan Ophthalmic Center (Sun Yat-sen University, Guangzhou, China) from January 2012 to September 2013. All clinical samples were from patients who were diagnosed with fungal keratitis and tested by culture and direct smear. Written informed consents was obtained from the participants or their guardians before the study, which conforms to the tenets of the Declaration of Helsinki. The controls were normal donor corneas remaining after corneal transplantation: a waiver of consent was given for these donor corneas, as they were obtained from an eye bank (Guangdong Eye Bank, Guangzhou, China). These samples were subjected to quantitative real-time PCR (qRT-PCR). This study was approved by the Institutional Review Board of the Zhongshan Ophthalmic Center (approval ID: 2012KYNL017).

### Cell lines and treatment

RAW264.7 macrophages (an immortalized cell line) were purchased from the American Type Culture Collection (Rockville, MD) and stored in a −80°C freezer. These cells were then thawed and cultured in DMEM with 10% heat-inactivated fetal bovine serum (Gibco-BRL, Gaithersburg, MD) and penicillin-streptomycin (Gibco-BRL, Gaithersburg, MD) in a culture flask at 37°C. Once they formed a sheet, the cells were also incubated with TrypLE (Gibco-BRL, Gaithersburg, MD). In total, 1×10^6^ of these RAW264.7 cells (passage 2–3) were pre-cultured in 1 ml of culture medium (described above) in a 12-well plate for 12 h before the following treatment.

The cells were divided into four groups of 1×10^6^ cells each: group I received zymosan (100 µg/ml), group II received zymosan (100 µg/ml) + mTREM-1/Fc protein (1 µg/ml), group III received zymosan (100 µg/ml) + FK506 (20 µM), and group IV was the control group and received no treatment.

### *Aspergillus fumigatus* spore preparation

The *Aspergillus fumigatus* strain used in this investigation was AS 3.772, and it was purchased from the China General Microbiological Culture Collection Center. The yeast strain was then grown on Sabouraud dextrose agar (Difco, Detroit, MI) at 30°C for 4 days. Spores were harvested and washed in sterile phosphate-buffered saline (PBS) and then diluted in sterile saline to a concentration of 10^6^ colony-forming units (CFU)/ml (*Aspergillus fumigatus* spore preparation, or AFSP).

### Murine model of *Aspergillus fumigatus* fungal keratitis

Six- to eight-week-old inbred female B6 mice were purchased from the Guangdong Provincial Center for Animal Research, Guangzhou, China. The mice were housed in a standard animal facility with a controlled temperature (22–24°C) and photoperiod (12 h light, 12 h dark) and were given free access to food and water. The animal experiments complied with the Association for Research in Vision and Ophthalmology Statement for the Use of Animals in Ophthalmic and Vision Research. The research protocol was also approved by the Animal Care Committee of the Zhongshan Ophthalmic Center at Sun Yat-sen University (Guangzhou, China) (approval ID: 20120308). The mice were anesthetized intraperitoneally with xylazine (1.9 mg/ml) and ketamine (37.5 mg/ml), and every effort was made to minimize suffering.

The intrastromal injection method [26] was used to establish the murine model of fungal keratitis. Briefly, the mice were anesthetized intraperitoneally with xylazine (1.9 mg/ml) and ketamine (37.5 mg/ml). A 30-gauge needle was then inserted into the right cornea of each mouse, near the center, to the depth of the superficial stroma. Next, a 33-gauge needle with a 30° bevel was threaded into the stroma to inject 2 µl AFSP. The *Aspergillus fumigatus*-infected mouse cornea exhibited obvious corneal edema, with stromal infiltration, at one day post-infection. All mice were examined using a slit-lamp microscope (Carl Zeiss Meditec, Dublin, CA), and those that did not meet this standard were excluded. In contrast, the mice with successfully induced fungal keratitis were randomly grouped and examined at 1, 3 and 5 days after infection. Corneal opacity and surface regularity were evaluated with a scoring system [27] to grade the severity of corneal damage. Additionally, images of the mouse corneas were acquired using a photo slit-lamp microscope (Carl Zeiss Meditec, Dublin, CA) with a digital camera (Nikon, Tokyo, Japan).

The mice with successfully induced fungal keratitis were randomly divided into 2 groups: a vehicle-treated group and an FK506-treated group. A 0.05% FK506 solution was supplied by Zhongshan Ophthalmic Center; the preparation of FK506 solution was described previously [28]. The formulation was a mixture of normal saline, hydrogenated castor oil (20% w/v), Tween-80 (5.5% v/v), hydroxymethyl cellulose (0.3% w/v), glycerol (8% v/v), and thiomersalate (0.002% w/v). The vehicle solution contained the same ingredients, except FK506.

The mice in the FK506-treated group were subconjunctivally injected with 5 µl of this FK506 solution 24 h before infection, after which the mice with infected eyes received FK506 solution 4 times per day for 5 consecutive days. Similarly, the mice in the vehicle group received treatment with vehicle eye drops. The animals were sacrificed at the indicated end points of the experiments by isoflurane anesthesia followed by cervical dislocation.

### Histologic examination

Eyes were fixed in 4% formaldehyde in 0.075 M phosphate buffer for 24 h, dehydrated in increasing concentrations of ethanol (70–99%) and infiltrated with paraffin (Merck, Darmstadt, Germany) at 60°C. Sections with a thickness of 5 µm were then cut and floated on deionized water at 45°C, and single sections were mounted on SuperFrost Plus glass slides (Menzel-Glaser, Braunschweig, Germany). The slides were subsequently dried at 60°C for 1 h. Hematoxylin and eosin (HE) staining was then performed to determine the degree of corneal edema, the level of inflammatory cell infiltration and corneal irregularity.

### *Aspergillus* colony-forming units (CFUs)

Whole corneas from the murine model of *Aspergillus fumigatus*-induced fungal keratitis were homogenized in 1 ml of sterile PBS. Subsequently, serial 10-fold dilutions were performed and plated onto Sabouraud dextrose agar plates (Merck, Darmstadt, Germany). The plates were then incubated at 37°C for 24 h, and the CFU number was determined by direct counting.

### ELISA

Each mouse cornea was homogenized in PBS containing 0.1% Tween-20. These homogenates were centrifuged at 12,000 g for 15 min to collect the supernatants. The protein concentration was then determined using the Quick Start Bradford protein assay (Bio-Rad, Hercules, CA). Additionally, ELISA kits (R&D Systems, Minneapolis, MN) were used to determine the protein levels of MPO, TREM-1, TNFα and IL-1β in the supernatants in triplicate according to the manufacturer’s instructions. The data were expressed as the amount of target molecule (picograms) per the amount of total protein (milligrams) in each sample. RAW264.7 cells (1×10^6^) were also seeded onto 12-well plates and administered different treatments as described above. Cell-free supernatants were collected after 24 h of cultivation, and sTREM-1, TNFα and IL-1β concentrations were determined using ELISA kits (R&D Systems).

### Quantitative real-time PCR

With a cataract knife, the cornea was perforated at the limbus, making an opening large enough to insert the tip of a pair of corneal scissors. Under an operating microscope, each whole cornea of each mouse was then carefully cut off through the corneal limbus with the corneal scissors. The total RNA in each cornea was extracted using the RNeasy Micro Kit (Qiagen Sciences, Germantown, USA) according to the manufacturer's protocol. Additionally, the total RNA in cells was extracted using TRIzol (Invitrogen, Carlsbad, CA) according to the manufacturer’s protocol. The RNA was quantified using a NanoDrop 2000C spectrophotometer (Thermo Scientific, West Palm Beach, USA) and reverse transcribed into cDNA using a kit (Fermentas, St. Leon-Rot, Germany). The primer sequences used were as follows: mouse glyceraldehyde-3-phosphate dehydrogenase (mGAPDH), 5′ CTT CCG TGT TCC TAC CCC 3′ (forward) and 5′ CCA AGA TGC CCT TCA GTG 3′ (reverse); mTREM-1, 5′ CTG TGC GTG TTC TTT GTC 3′(forward) and 5′ CTT CCC GTC TGG TAG TCT 3′(reverse); hGAPDH, 5′ AAG GTG AAG GTC GGA GTC 3′(forward) and 5′ CAA GCT TCC CGT TCT CAG C 3′(reverse); hTREM-1, 5′ AGC CAG AAA GCT TGG CAG A 3′(forward) and 5′ TGG AGA CAT CGG CAG TTG 3′(reverse); mTNFα, 5′ GGT CAA TCT GCC CAA GTA 3′(forward) and; 5′ GCC ATA ATC CCC TTT CTA 3′ (reverse) and mIL-1β, 5′ TGT TTT CCT CCT TGC CTC T 3′ (forward) and 5′ TGC CTA ATG TCC CCT TGA 3′(reverse). SYBR Green Master Mix (Bio-Rad, Hercules, CA) and the CFX96 Real-Time PCR Detection System (Bio-Rad, Hercules, CA) were used to perform qRT-PCR analysis according to the instructions provided by the manufacturer. The data were analyzed according to the comparative Ct (ΔΔCT) method and were normalized to GAPDH expression in each sample.

### Statistical analysis

An unpaired t test was performed to determine the differences in the qRT-PCR, ELISA, and fungal viability results. The clinical scores were reported as the mean ± SEM, and a Mann-Whitney U test was used to determine the significance of differences between the vehicle-treated group and the FK506-treated group. A P-value less than 0.05 was considered significant.

## Results

### TREM-1 expression was higher in the corneas of fungal keratitis patients than in normal human corneas

**Figure 1:**
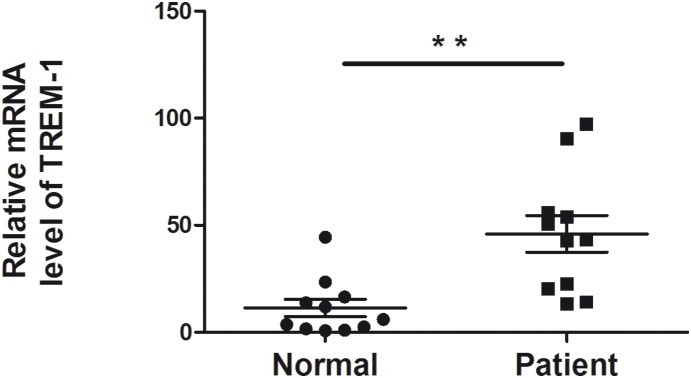
TREM-1 expression in human corneas. TREM-1 mRNA levels were examined in normal human corneas and human corneas with fungal keratitis. The data are the mean ± SEM, with 11 patients/group (**P<0.01).

TREM-1 expression was detected in fungus-infected corneas and normal human corneas to determine whether TREM-1 induces corneal inflammation. In fact, TREM-1 was significantly increased in fungus-infected corneas, as shown by the qRT-PCR data (Fig. 1).

### Zymosan induced RAW264.7 cells to produce TREM-1 and proinflammatory cytokines in a dose-dependent manner

**Figure 2:**
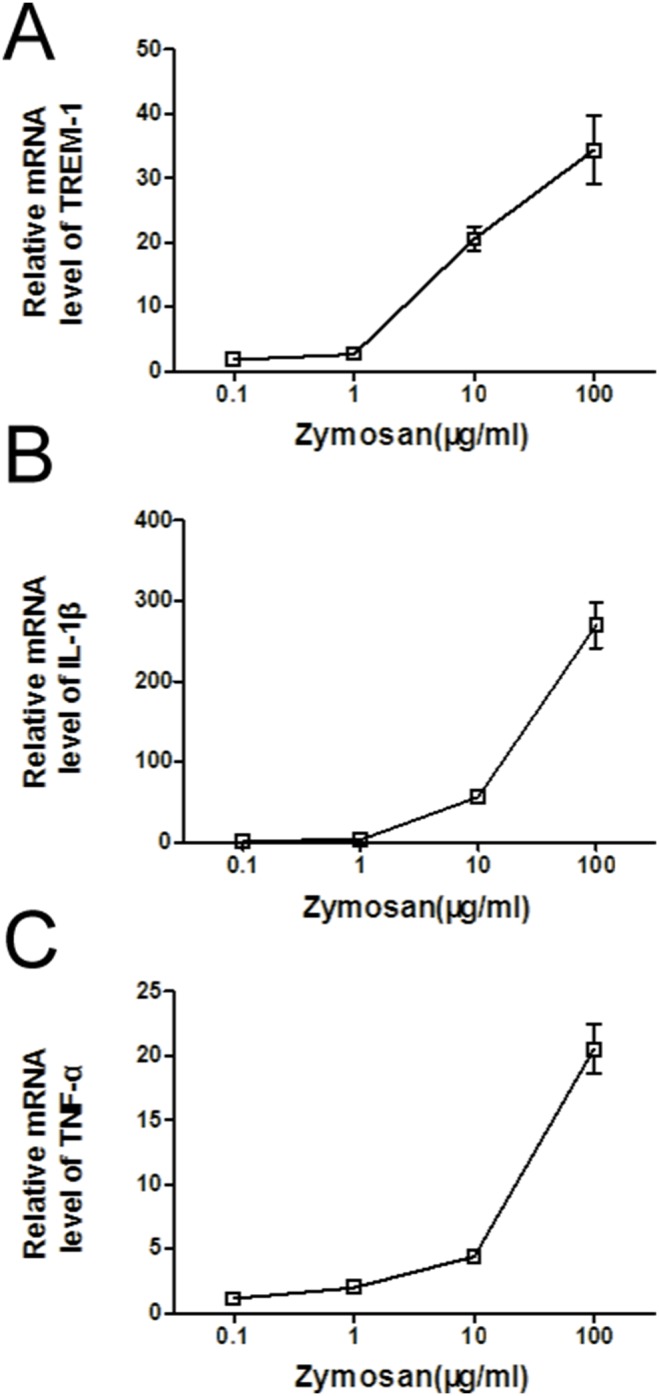
TREM-1, TNFα, and IL-1β mRNA expression in RAW264.7 cells after zymosan stimulation. TREM-1 mRNA expression (A), IL-1β mRNA expression (B) and TNFα mRNA expression (C) stimulated by zymosan (0.1–100 µg/ml) for 8 h.

To further investigate the effect of TREM-1 on innate immunity, we stimulated RAW264.7 cells with zymosan, a component of the fungal cell wall. The mRNA expression levels of TREM-1 (Fig. 2A), IL-1β (Fig. 2B) and TNFα (Fig. 2C) were gradually enhanced in a dose-dependent manner and peaked at a concentration of 100 µg/ml.

### TREM-1 expression increased after zymosan treatment in a mouse macrophage cell line (RAW264.7 cell line)

**Figure 3:**
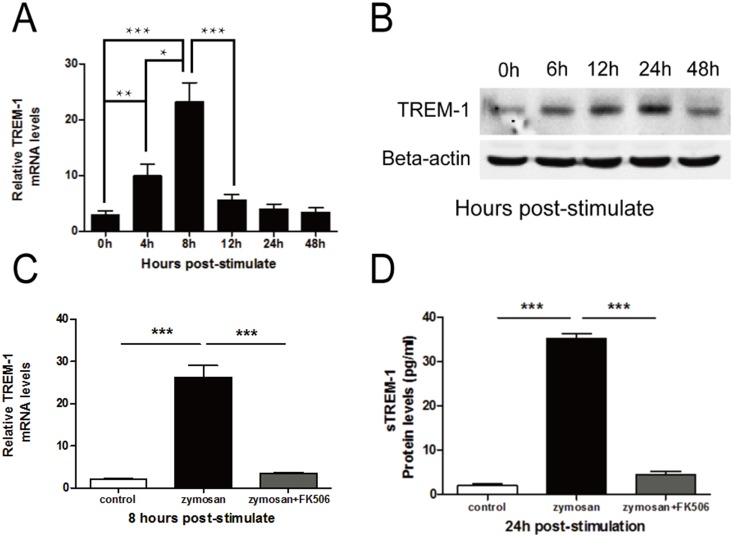
The effect of FK506 on TREM-1 expression in RAW264.7 cells. The mRNA (A) and protein (B) expression of TREM-1 was determined at different time points after zymosan (100 µg/ml) stimulation. RT-PCR data (C) indicated that FK506 downregulated TREM-1 expression at 8 h post-stimulation. ELISA data (D) demonstrated that sTREM-1 protein expression decreased at 24 h post-stimulation. The data are presented as the mean ± SEM and represent 3 individual experiments, with 5 groups of cells at each time point (***P<0.001).

TREM-1 expression in RAW264.7 cells changed in a time-dependent manner. The data also showed that TREM-1 mRNA expression increased at 4 h and peaked at 8 h after stimulation (Fig. 3A), whereas the protein expression of TREM-1 increased at 6 h and peaked at 24 h after stimulation (Fig. 3B).

### TREM-1 expression greatly decreased in RAW264.7 cells after FK506 treatment

To determine whether FK506 can affect TREM-1 expression, the mRNA and protein levels of TREM-1 were measured in the FK506-treated RAW264.7 cells and the control group. The PCR data (Fig. 3C) and ELISA data (Fig. 3D) indicated that TREM-1 expression was significantly decreased in FK506-treated RAW264.7 cells compared with the control cells after stimulation with zymosan.

### FK506 decreased the expression of inflammatory factors in RAW264.7 cells after zymosan stimulation

**Figure 4:**
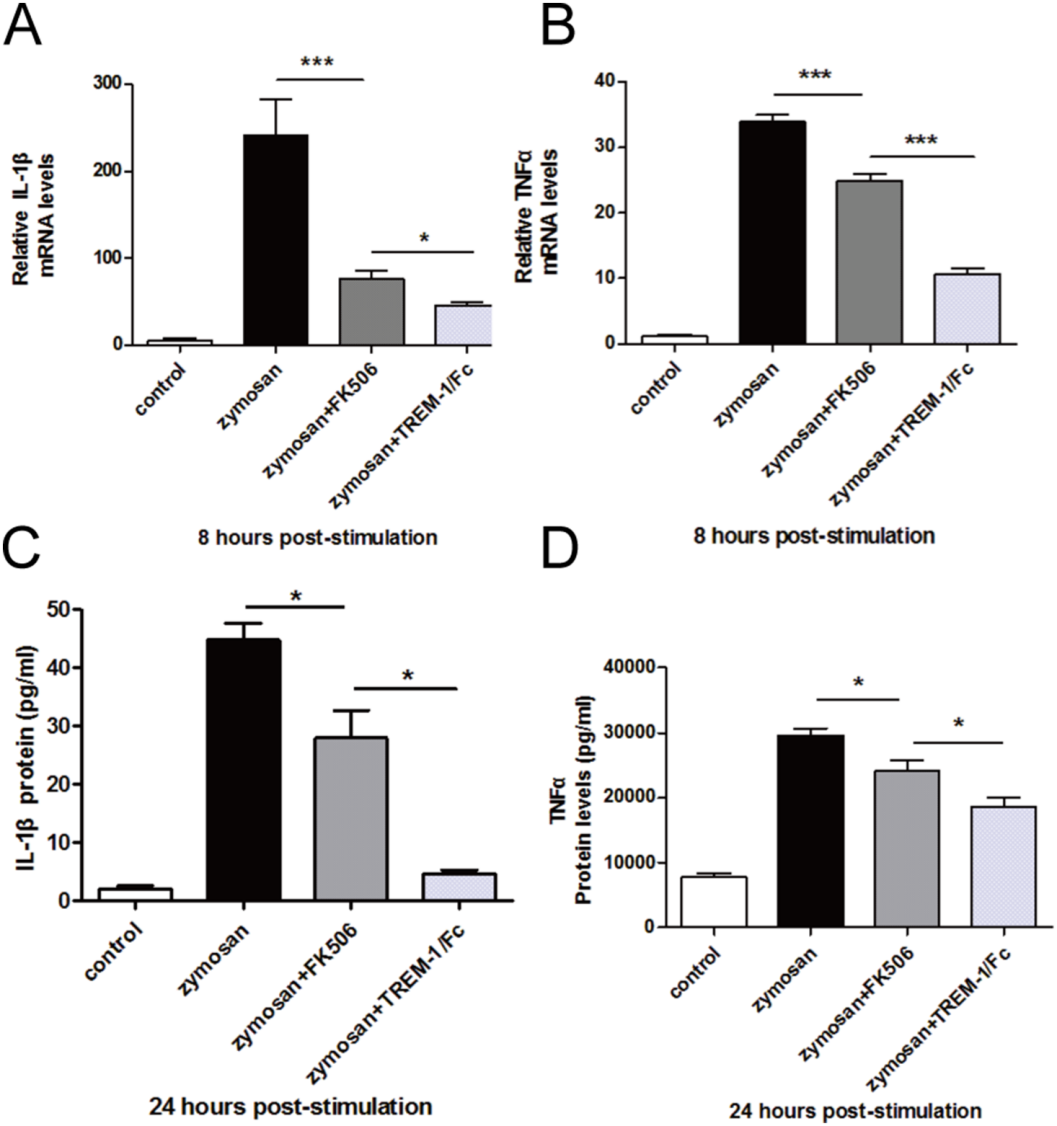
Inflammatory cytokine production after treatment with FK506 in vitro. RT-PCR data demonstrated that at 8 h post-stimulation, the expression of IL-1β (A) and TNFα (B) was downregulated. ELISA data (C and D) also indicated that FK506 significantly reduced inflammatory cytokine protein levels at 24 h post-stimulation. The data are presented as the mean ± SEM and represent 3 individual experiments, with 5 groups of cells at each time point (*P<0.05; ***P<0.001).

To evaluate the effects of the FK506-mediated downregulation of TREM-1 expression, we analyzed the expression of IL-1β and TNFα. These two inflammatory factors are both downstream of TREM-1 [29], [30]. We used TREM-1/Fc-treated cells as positive controls. Compared with zymosan, alone FK506 significantly reduced the expression of IL-1β and TNFα at both the mRNA (Fig. 4A, Fig. 4B) and the protein (Fig. 4C, Fig. 4D) levels.

### FK506 reduced corneal damage at an early stage after *Aspergillus fumigatus* infection

**Figure 5:**
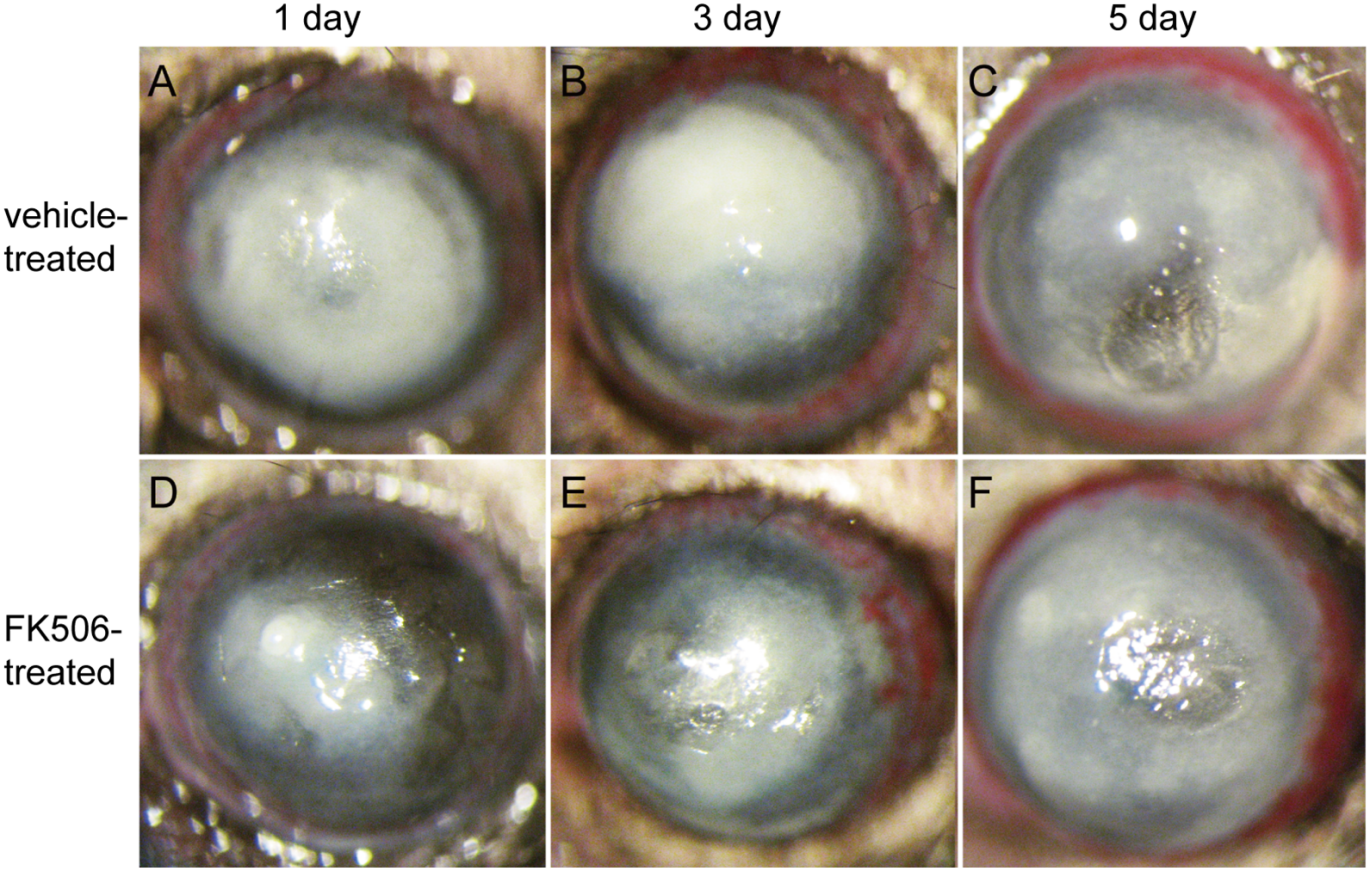
Clinical progression of B6 mice after *Aspergillus fumigatus* infection. The images in the first row (A, B and C) show the pathologic process in vehicle-treated B6 mice (n = 5/group/time point) at 1, 3 and 5 days post-infection The second row (D, E and F) shows the appearance of the FK506-treated group (n = 5/group/time) at the same time points. The images from day 1 post-infection show uniform opacity and significant swelling of the corneas from vehicle-treated B6 mice. In contrast, there was less cloudiness, an outline of the iris and slight surface irregularity in the FK506-treated corneas, indicating that corneal damage was greatly reduced at 1 day post-infection.

**Figure 6:**
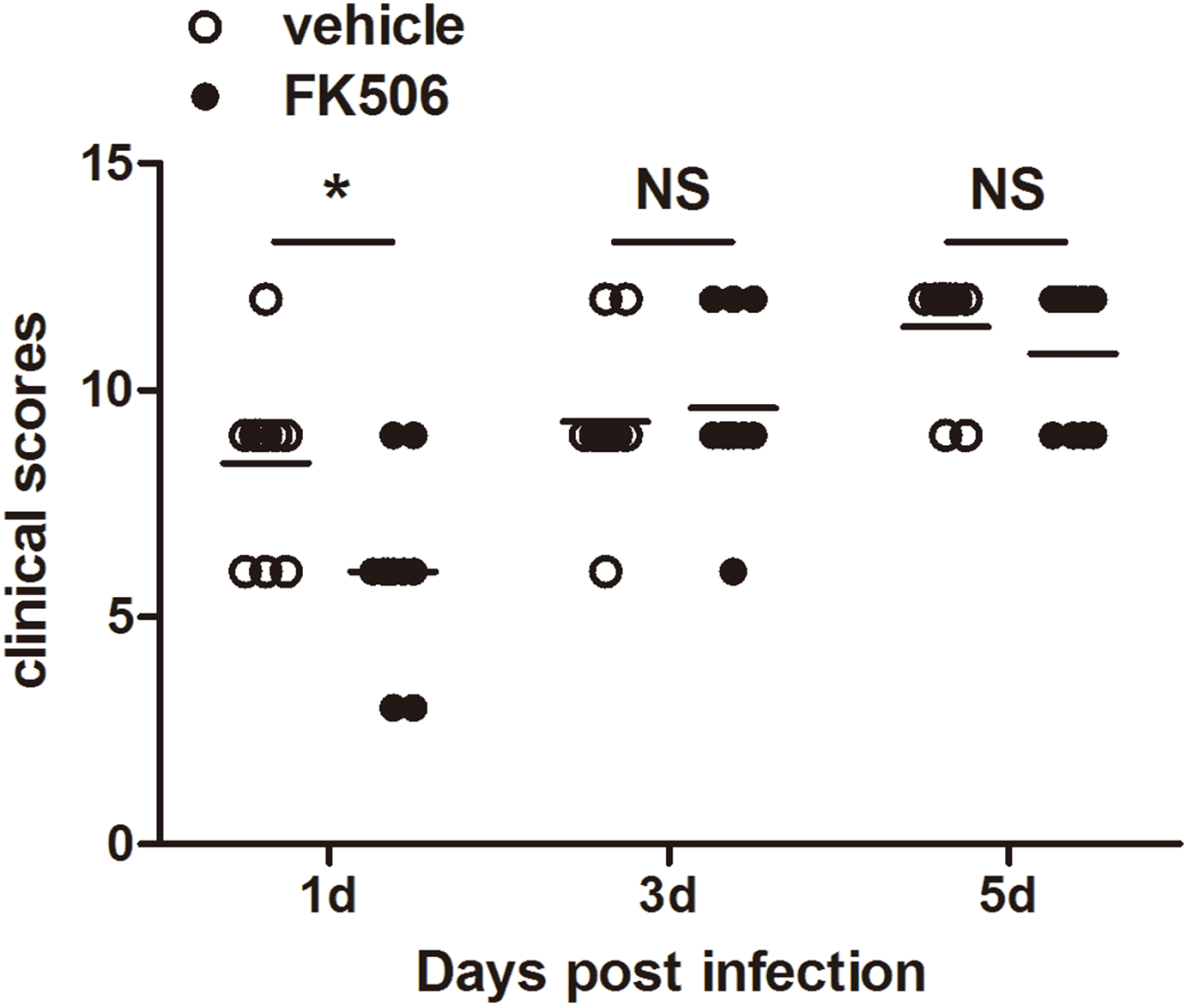
FK506 promoted host resistance to fungal infection at an early stage. Corneal clinical scores decreased after treatment with FK506 at 1 day post-infection, but no difference was detected at 3 and 5 days post-infection (*P<0.05).

**Figure 7:**
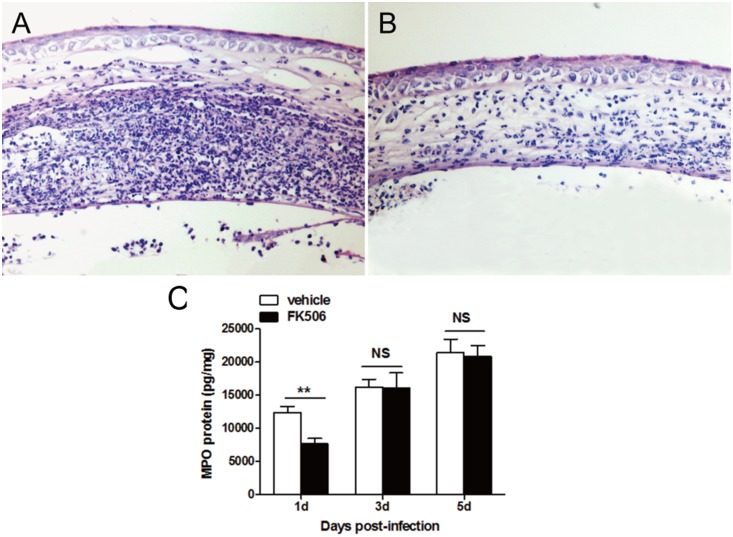
FK506 suppressed PMN infiltration in mouse corneas. Histopathology of infected corneas at 1 day post-infection There was higher PMN infiltration and corneal edema in group A (A) than in group B (B). PMN infiltration was measured using an MPO ELISA (C) (**P<0.01).

Because TREM-1 expression levels were significantly decreased after treatment with FK506 in vitro, the subsequent series of experiments was designed to determine whether FK506 could reduce ocular disease after Aspergillus fumigatus infection. In particular, B6 mice were subconjunctivally injected and topically treated with FK506 or vehicle. The slit-lamp photographs in Fig. 5 depict representative mouse eyes at 1, 3 and 5 days post-infection We also assessed the clinical scores (Fig. 6) of ocular disease post-infection These clinical scores indicated that FK506-treated mice displayed minor damage at 1 day post-infection (*P*<0.05), but there was no significant difference between the two groups at 3 or 5 days post-infection. Moreover, HE staining indicated that there was greater polymorphonuclear neutrophil (PMN) infiltration and corneal edema in the vehicle-treated mice (Fig. 7A) than in the FK506-treated mice (Fig. 7B). MPO is a peroxidase enzyme that is most abundantly expressed in the cytoplasmic granules of neutrophils. Therefore, the expression levels of MPO are a direct measure of neutrophil sequestration in tissue [31]. To elucidate the mechanism through which FK506 modulates the immune response against fungal infection, PMN infiltration was evaluated via an MPO protein concentration assay (Fig. 7C). The ELISA data indicated that MPO expression was reduced in the FK506-treated group at 1 day post-infection (*P*<0.001), but no significant differences were detected between the two groups at 3 or 5 days post-infection.

### Fungal viability was increased in mouse corneas at an early stage after treatment with FK506

**Figure 8:**
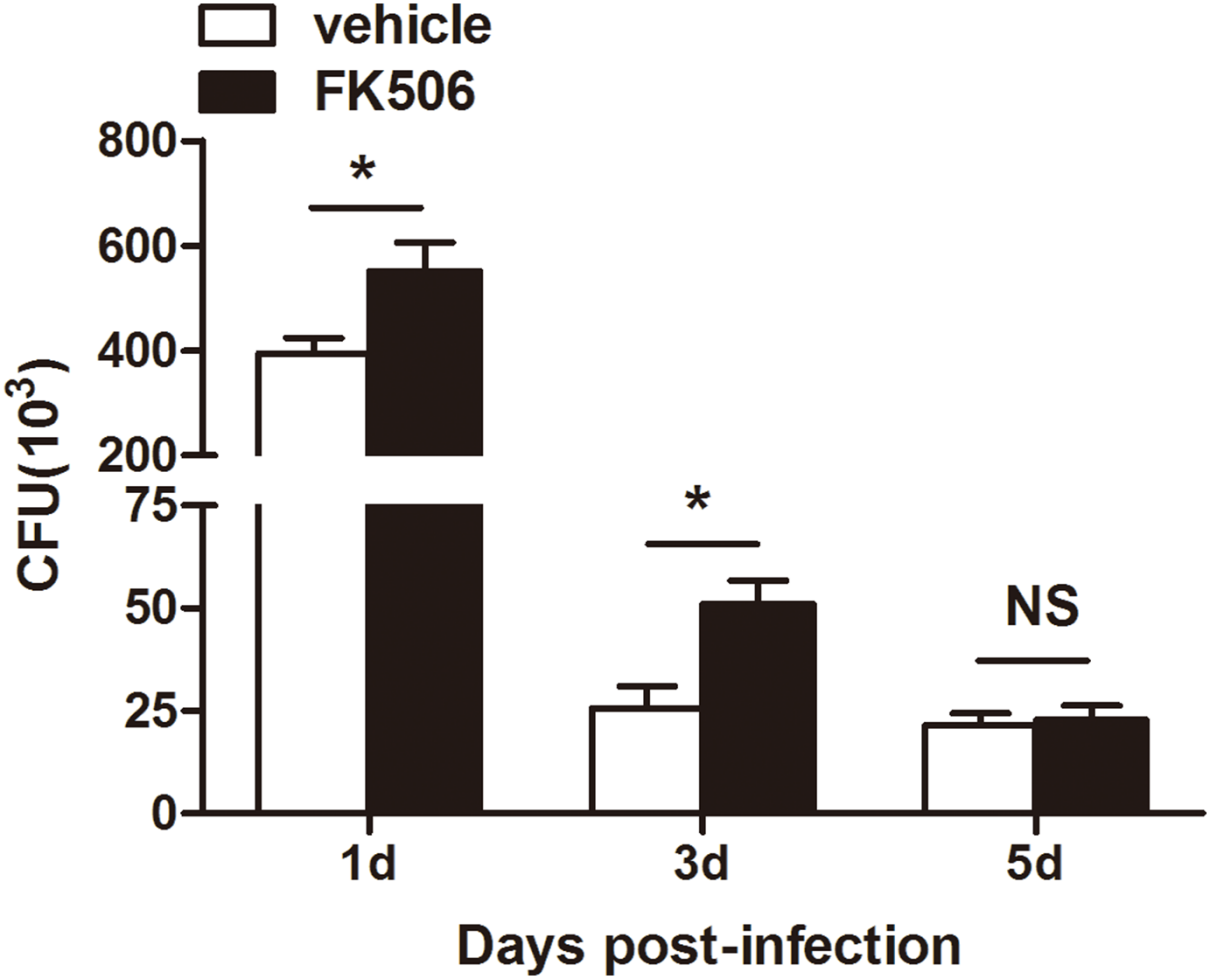
Fungal viability in a fungal keratitis model. Fungal viability was determined via fungal plate counts *P<0.05).

To elucidate the mechanism by which FK506 modulates the immune response against fungal infection, fungal viability was determined via fungal plate counts (Fig. 8). The CFU data indicated a significant increase in the FK506-treated group at 1 and 3 days post-infection (Fig. 8, both *P*<0.05), but there was no significant difference at 5 days post-infection (*P* = 0.795).

### FK506 reduced the early expression of TREM-1 after fungal infection *in* *vivo*

**Figure 9:**
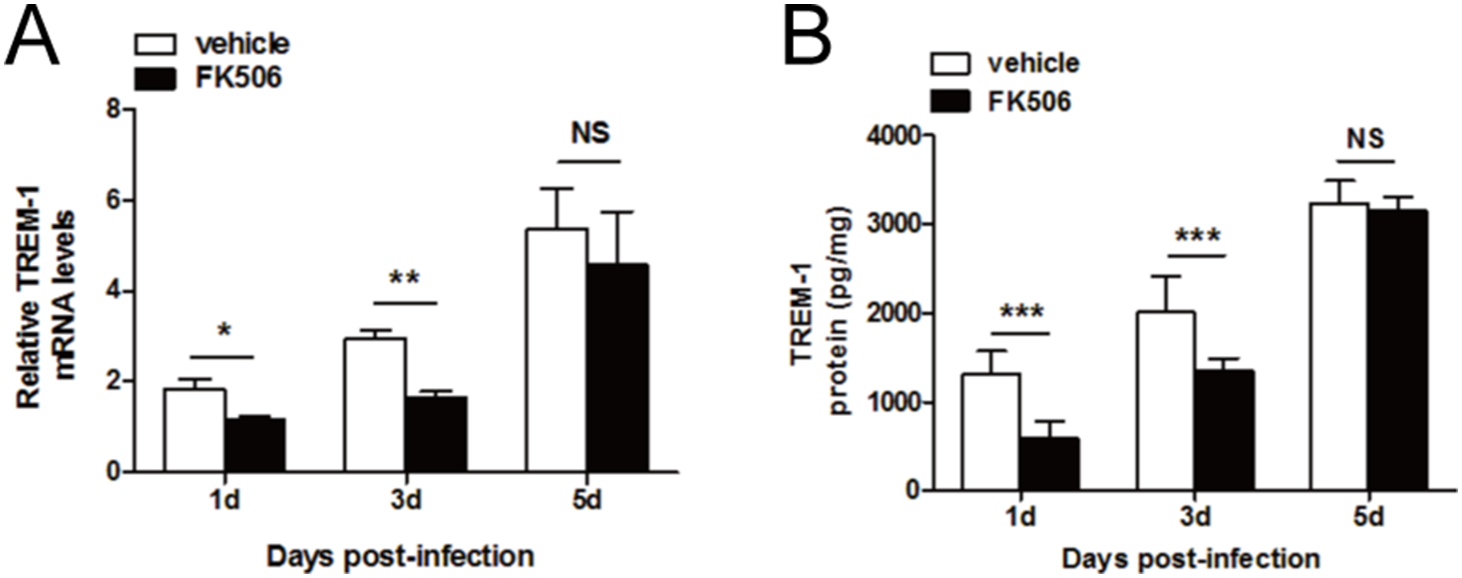
TREM-1 expression in B6 mice after treatment with FK506. RT-PCR data (A) indicated that at 1 and 3 days post-infection, treatment with FK506 downregulated TREM-1 expression. ELISA data (B) demonstrated that at 1 and 3 days post-infection, TREM-1 protein expression decreased after treatment with FK506, whereas no change was detected between the vehicle-treated group and the FK506-treated group at 5 days post-infection. The data are presented as the mean ± SEM and represent 3 individual experiments, each with 5 animals at each time point (*P<0.05; **P<0.01; ***P<0.001).

To further determine the role of FK506 in the regulation of TREM-1 expression, we analyzed TREM-1 mRNA (Fig. 9A) and protein (Fig. 9B) expression levels. There was a decrease in TREM-1 expression at both levels at 1 day post-infection (*P*<0.05), similar to the clinical score data. However, in contrast to the clinical scores, there was a decrease in TREM-1 expression at both levels at 3 day post-infection (*P*<0.01), whereas no change was detected in either TREM-1 expression levels or the clinical score data between the two groups at 5 days post-infection.

### FK506 reduced the early expression of inflammatory factors in fungus-infected mice corneas

**Figure 10:**
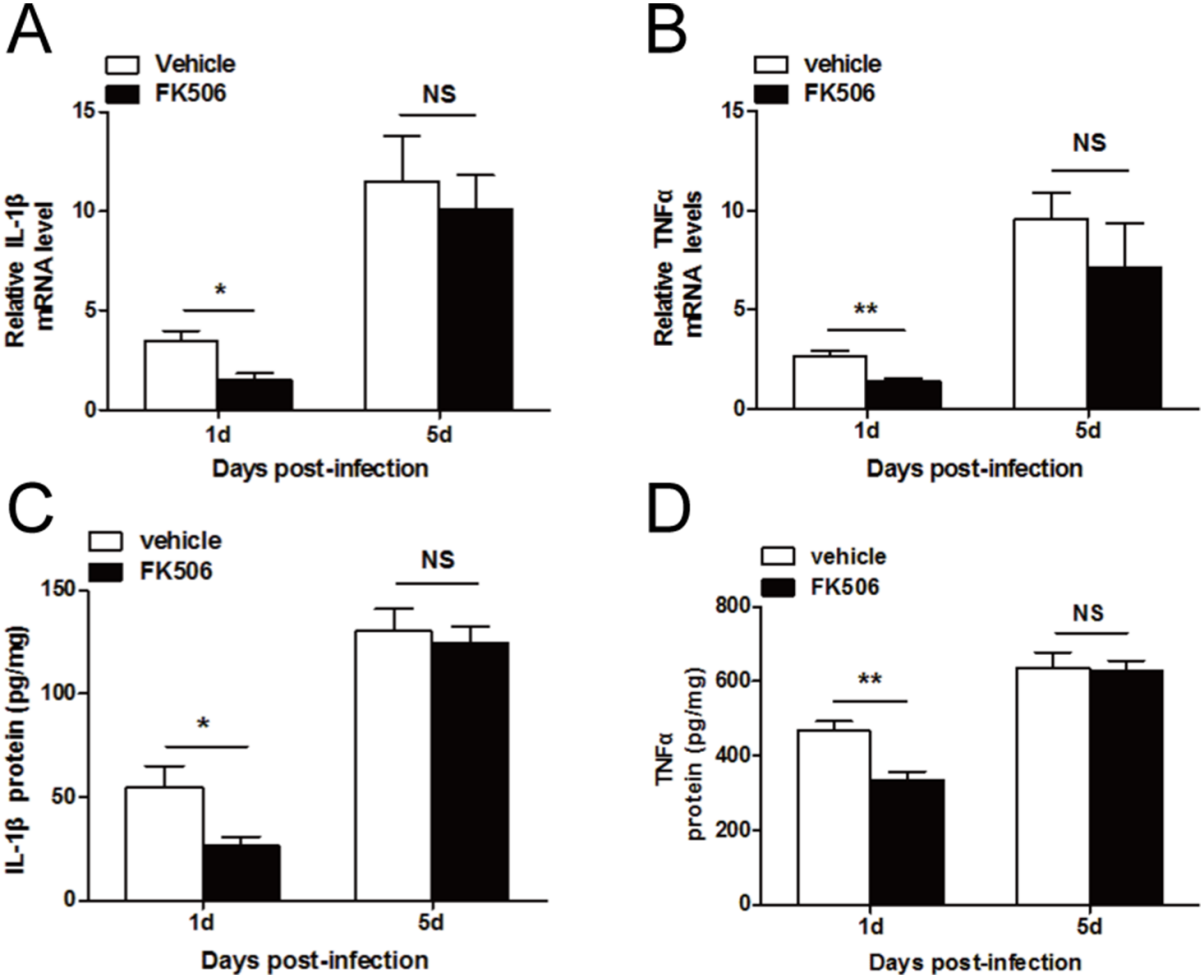
Inflammatory cytokine production in mouse corneas after treatment with FK506. RT-PCR (A) and ELISA (C) data demonstrated that at 1 day and 3 days post-infection, IL-1β expression was downregulated in the FK506-treated group, but no differences were found at 5 days post-infection. The RT-PCR (B) and ELISA (D) data also indicated that FK506 significantly reduced TNFα expression at 1 day post-infection, but no differences between the two groups were observed at 5 days post-infection The data are presented as the mean ± SEM and represent 3 individual experiments, with 5 animals at each time point (*P<0.05; **P<0.01).

To determine the effect of the modulation of the immune response by FK506, we examined the expression of two inflammatory factors, IL-1β and TNFα (mRNA and protein). The RT-PCR (Fig. 10A) and ELISA (Fig. 10C) data indicated that FK506-treated mice exhibited a reduced expression level of IL-1β at 1 day post-infection (*P*<0.05, and *P*<0.05, respectively). Furthermore, the data indicated decreased TNFα expression in the FK506-treated group at both the gene (Fig. 10B) and the protein (Fig. 10D) levels at 1 day post-infection (*P*<0.01, and *P*<0.01, respectively). However, no differences were found at 5 days post-infection.

## Discussion

Fungal keratitis is a type of serious suppurative ocular disease [5] that can lead to extensive vision loss. Although appropriate therapy can eliminate the fungal load, corneal scarring can still cause blindness. Given that excessive inflammation is an important contributor to corneal damage during fungal infection [7], it is essential to attenuate the inflammatory response during fungal infection. This inflammatory response of the immune system is a double-edged sword in the host defense mechanism. On the one hand, this response is the main approach that the immune system uses to eliminate pathogenic fungi, which is beneficial in protection against fungal infection. On the other hand, overenthusiastic inflammation can be damaging, and persistent inflammation can lead to degeneration or necrosis of tissue [32]. Therefore, it is essential to attenuate the inflammatory response during fungal infection.

As an amplifier of inflammation, TREM-1 expression is dramatically increased by exposure to bacteria and fungi. Studies have indicated that proinflammatory cytokines, such as TNFα and IL-1β, are in turn upregulated when TREM-1 is activated in the presence of TLR2 or TLR4 ligands [8]. Additionally, experiments in a mouse model of septic shock established that blocking TREM-1 downregulated the plasma concentrations of TNFα and IL-1β, reduced monocyte/macrophage infiltration into the peritoneum, and partially protected animals from death [6], [33]. The studies cited above confirmed that TREM-1 serves as an amplifier of inflammation and plays an important role in infectious disease. In the present study, we first demonstrated that TREM-1 expression was greatly upregulated in *Aspergillus fumigatus*-infected human corneas compared with uninfected human corneas. TREM-1 expression was then found to be upregulated in a murine macrophage cell line (RAW264.7 cell line) after stimulation with zymosan, a fungal cell wall particle that has often been used as a mimic of fungal stimulation of the innate immune system [34]. This finding indicated that there is a potentially close relationship between TREM-1 and fungal keratitis.

The most widely used anti-inflammatory agents include corticosteroids, non-steroidal anti-inflammatory drugs and CsA. However, there are obvious disadvantages to all of the anti-inflammatory agents listed above. For example, corticosteroids have a strongly inhibitory effect on inflammation, but the side effects of topical steroids also include cataract formation and a rise in intraocular pressure [35]. Furthermore, studies have indicated that topically applied corticosteroids accelerate the speed of invasion of fungi, so these drugs are forbidden for the treatment of active fungal keratitis [36]. Meanwhile, non-steroidal anti-inflammatory drugs have an effect on prostaglandins, which are only a minor part of inflammation in fungal keratitis. However, non-steroidal anti-inflammatory drugs may also induce keratitis, ulceration, and perforation [37]. Thus, topical immunosuppressants may be a safer choice. Growing evidence indicates that macrolides inhibit the inflammatory activities of the innate and adaptive immune systems. Although hypotheses have been proposed to provide an explanation for this anti-inflammatory effect, it is believed that the anti-inflammatory effect is due to inhibition of the nuclear translocation of nuclear factor-kB (NF-kB) and activator protein-1 (AP-1) by macrolides [38], [39].

FK506 is a macrolide antibiotic with immunosuppressive properties that is produced by *Streptomyces tsukubaensis*. A target of FK506 and CsA, calcineurin (cnaA) is important for *Aspergillus fumigatus* growth, morphology, and pathogenicity. Therefore, a mutant *Aspergillus fumigatus* strain without the cnaA catalytic subunit presents physiological defects that critically affect the fitness of the fungus and lead to stunted growth [40]. A broth susceptibility test of *Aspergillus fumigatus* also demonstrated that *Aspergillus fumigatus* growth was inhibited after FK506 treatment [40]. These studies indicated that cnaA inhibitors play a role in inhibiting fungal growth.

In the current study we found that FK506 inhibits inflammation without affecting fungal growth in fungal keratitis. Many researchers have shown that an important application of FK506 is as a drug for effectively inhibiting the inflammatory process. In particular, recent studies have indicated that FK506 demonstrates efficacy in the treatment of many types of ocular diseases, including corneal graft rejection [38], [39], vernal keratoconjunctivitis [41], atopic keratoconjunctivitis [42], and uveitis [43]. Additional investigations have demonstrated that the possible mechanism of FK506 in the treatment of ocular diseases may involve the ability of FK506 to reduce T-lymphocyte activation [44], [45] and to downregulate the expression of inflammatory response-related genes [41].

Although the inhibitory mechanisms of FK506 have been extensively studied in T cells, little is known about the precise suppressive mechanisms of FK506 in non-T cells. In the present study, FK506 exerted an obvious anti-inflammatory effect not only in a cell model of fungal infection mimicked by stimulation with zymosan, but also in a mouse model of fungal keratitis induced by *Aspergillus fumigatus*. We found that FK506 may reduce the infiltration of inflammatory cells by suppressing the expression of proinflammatory cytokines such as TNFα and IL-1β and downregulating the expression of TREM-1 at an early stage of fungal infection in corneas.

The anti-inflammatory effects of FK506 most likely rely on several molecular mechanisms: (1) FK506 prevents the activation of cnaA, which in turn inhibits the dephosphorylation of nuclear factor of activated T cells (NFAT), a transcription factor that plays a significant role in activating the genes encoding cytokines involved in the regulation of an immune response, such as IL-2 [46]. (2) FK506 reduces the transcriptional activation of AP-1 and NF-kB, factors that are linked to the activation of early cytokine genes [47], [48]. (3) FK506 has been shown to suppress the APP synthesis induced by prostaglandins during injury or inflammation [49]. (4) FK506 dose-dependently decreases MPO activity in inflamed tissue, demonstrating the capacity of FK506 to suppress neutrophil migration to inflammatory tissues.

In conclusion, FK506 was used to inhibit the overenthusiastic inflammation induced by fungi in this study. The results indicated that FK506 significantly reduced TREM-1 expression and the release of inflammatory cytokines at an early stage of fungal infection. Notably, inhibition of TREM-1 is not effective enough to completely clear fungi form the cornea. The reason is that although FK506 has a strong inhibitory effect on the inflammation induced by the fungal antigens, it may weaken the elimination of fungi by inhibiting the activation of inflammatory cells. FK506 may inhibit the inflammation induced by fungi and alleviat the severity of corneal damage at an early stage of fungal keratitis by downregulating TREM-1 expression, so future research on treatments for fungal keratitis will hopefully enable the development of antifungal drugs that can be combined with FK506.
